# Four Types of ST11 Novel Mutations From Increasing Carbapenem-Resistant *Klebsiella pneumoniae* in Guangdong, 2016–2020

**DOI:** 10.3389/fmicb.2021.702941

**Published:** 2021-10-01

**Authors:** Yunhu Zhao, Yalong Liao, Ni Zhang, Suling Liu, Jiao Zhang, Xuejiao Hu, Dianrong Zhou, Qianyun Deng, Yanping Shi, Bing Gu, Tieying Hou

**Affiliations:** ^1^Guangdong Provincial People’s Hospital, Guangdong Academy of Medical Sciences, Guangzhou, China; ^2^Dermatology Hospital of Southern Medical University, Guangzhou, China; ^3^General Hospital of Southern Theatre Command, Guangzhou, China

**Keywords:** *Klebsiella pneumonia*, carbapenemase, antibacterial susceptibility, MLST, ST11 mutation

## Abstract

**Objectives:** This study aimed to explore changes in carbapenem-resistant *Klebsiella pneumoniae* (CR-KP) isolates collected in Guangdong over the period of 2016–2020.

**Methods:** Antibacterial susceptibility was quantified through VITEK 2 compact and K-B method. Carbapenemase phenotypes and genotypes were characterized by modified carbapenem inactivation method (mCIM), EDTA-carbapenem inactivation method (eCIM), and polymerase chain reaction (PCR). Molecular characteristics and evolutionary trends were analyzed by multilocus sequence typing and evolutionary tree.

**Results:** Isolates (2,847) of *K. pneumoniae* were separated in 2016–2020, and the separate rate of CR-KP increased from 5.65 to 9.90% (*p* = 0.009). The top 3 wards were intensive care unit (ICU) (21.92%), neonatal wards (13.70%), and respiratory wards (12.33%). In 146 CR-KP strains, serine carbapenemase was the main phenotype, and KPC was the main genotype, and 57 contained two resistant genes, and 1 contained three resistant genes. Two polygenic strains were first found: IMP + GES and KPC + NDM + VIM, but all the phenotypes were metalloenzyme, which indicated that metalloenzyme was usually the first choice for CR-KP resistance. In addition, all the ST54 of metalloenzyme type contained IMP, and all the ST45, ST37, and ST76 contained OXA. ST11 was the most prevalent (42.47%); ST11 and its mutants proved the predominant sequence type making up 51.1% of the carbapenemase-producing isolates. A novel type of ST11 mutation, the *rpoB* was mutated from sequence 1 to sequence 146, was in an independent separate branch on the evolutionary tree and was resistant to all antibacterial agents. The other three mutants, *rpoB 1*–15, *infB* 3–148, and *infB* 3–80, are also resistant to all antibacteria. Of note, all the four mutants produced serine carbapenemase and contained KPC, and indicated that the prevalent strain in China, ST11, has serious consequences and potential outbreaks.

**Conclusion:** The infection rate of CR-KP has increased, and ICU and neonatal wards have become the key infection areas. Producing serine enzyme, the KPC genotype, and ST11 are the predominant CR-KP. Polygenic strains and ST11 mutation made clinical treatment difficult and may become a potential threat.

## Introduction

*Klebsiella pneumoniae*, a conditionally pathogenic bacterial species, mainly causes community- and hospital-related infections (Gomes et al., 2021). The number of multi-drug-resistant (MDR) *K. pneumoniae* strains has gradually increased due to the wide application of broad-spectrum antibacterial agents. The increase in the prevalence of carbapenem-resistant *K. pneumoniae* (CR-KP) has become a serious public health problem (Yang et al., 2021). The World Health Organization and US Centers for Disease Control and Prevention (CDC) indicated that MDR *K. pneumoniae* is an immediate threat to human health (Centers for Disease Control Prevention, 2013; World Health Organization, 2017). According to the China Antimicrobial Resistance Surveillance System (CARSS), the detection rate of CR-KP in China increased from 6.4 to 10.1%, from 2014 to 2018 (China Antimicrobial Resistance Surveillance System, 2020); as such, CR-KP has been widely disseminated in China.

The mechanism of CR-KP is complex and may be caused by one or more factors, especially the production of carbapenemase (Gomes et al., 2021; Indrajith et al., 2021). When *K. pneumoniae* obtains carbapenemase, it becomes resistant to most β-lactam antibiotics, including carbapenem. In 2018, the Clinical and Laboratory Standards Institute (CLSI) updated the modified carbapenem inactivation method (mCIM) and the EDTA-carbapenem inactivation method (eCIM) to screen carbapenemase-producing strains and distinguish among carbapenemase types (Clinical Laboratory Standards Institute, 2018). CR-KP is classified into three types: carbapenemase negative, serine carbapenemase, and metallo-β-lactamase. At the molecular level, the main determinants of the enzyme-producing genotype include KPC, GES, NDM, IMP, VIM, and OXA, and the production of KPC is the most common in CR-KP (Calderaro et al., 2021; Indrajith et al., 2021). Therefore, CR-KP has received clinical attention because CR-KP infection has limited treatment options and high mortality.

The spread of CR-KP has gradually increased and even led to outbreaks (Kizilay et al., 2020; Pellicé et al., 2021). Molecular epidemiology plays an important role in tracking the spread of MDR bacteria to provide data for controlling their dissemination (Fontana et al., 2020). Methods for studying the molecular epidemiology of CR-KP include multilocus sequence typing (MLST), pulsed-field gel electrophoresis, enterobacterial repetitive intergenic consensus–polymerase chain reaction, and metagenomics next-generation sequencing. MLST is very mature and has a global standardized database; this method has good repeatability and high resolution and is easily operated, economical, affordable, and suitable for long-term epidemiological investigation (Institut Pasteur MLST and Whole Genome MLST Databases, 2016). At present, Asia is dominated by sequence type 11 (ST11), while Europe and America are dominated by ST258; these strains are closely related (Li et al., 2019). In addition, different types of CR-KP have different antibacterial profiles, virulence, pathogenicity, transmission, and evolutionary trends (Li et al., 2019; Gomes et al., 2021; Hassoun-Kheir et al., 2021; Huang et al., 2021). The distribution of ST11 and ST258 is accompanied by broad-spectrum drug resistance and high virulence, and CR-KP is increasingly regarded as a potential superbug.

In this study, we analyzed the distribution and antimicrobial susceptibility of CR-KP in Guangdong Provincial People’s Hospital over the period of 2016–2020 through mCIM, eCIM, PCR, and MLST to explore carbapenemase types, resistance mechanism, molecular epidemiology, and evolutionary trends. Results provide a theoretical basis for rational use of antibacterial agents, optimize treatment options, and control the spread of CR-KP.

## Materials and Methods

### Isolation and Culture of Carbapenem-Resistant *K. pneumoniae*

All isolates were obtained from Guangdong Provincial People’s Hospital, Dermatology Hospital of Southern Medical University, and General Hospital of Southern Theatre Command over the period of 2016–2020. Clinical samples were isolated and cultured on blood agar and mac agar plate. The target strain was identified by VITEK MS systems and tested for antimicrobial susceptibility by VITEK 2 compact and K-B method.

CR-KP is resistant to one of the carbapenems (imipenem/MIC ≥ 4 μg/ml, meropenem/MIC ≥ 4 μg/ml, or ertapenem/MIC ≥ 2 μg/ml), then the duplication was removed, and the first strain was reserved. Culture and preservation conditions: 5% CO_2_ at 35°C for 22–24 h and frozen with 20% glycerol at −70°C. One hundred forty-six carbapenemase-resistant strains were successfully recovered from a collection of 237 strains, and these CR-KP were not artificially selected but were included in the group based on the antimicrobial susceptibility results.

### Carbapenemase Phenotypic Tests

According to the 2018 CLSI update, mCIM and eCIM were used to detect the type of enzymes (Clinical Laboratory Standards Institute, 2018), which can be divided into three types, namely, carbapenemase negative, serine carbapenemase, and metallo-β-lactamase. ATCC BAA1705 and 1706 were used as the positive control and negative control in carbapenemase phenotypes, respectively.

### Extraction of DNA

The frozen strains were resuscitated and cleaved with 1% NP40 to obtain DNA, which was stored at −20°C (Qin et al., 2019).

### Detection of Resistance Genes

Six carbapenem-resistant genes were detected by PCR: KPC, GES, NDM, IMP, VIM, and OXA. The involved primers are shown in Table 1. The conditions were as follows: pre-denaturation at 95°C for 5 min; 35 cycles of 95°C for 30 s, 55°C for 30 s, and 72°C for 50 s; and extension at 72°C for 10 min. The PCR products were identified by agarose gel electrophoresis. *K. pneumoniae* contained KPC or KPC + NDM, and *E. coli* contained NDM, which were used as a control in carbapenemase genotypes.

**TABLE 1:** Oligonucleotide primers used for polymerase chain reaction (PCR).

Gene	Primer	Sequence (5′→3′)	Product size (bp)
bla_*KPc*_	KPC-F	TCGCTAAACTCGAACAGG	785
	KPC-R	TTACTGCCCGTTGACGCCCAATCC	
bla_*oEs*_	GES-F	CTATTACTGGCAGGGATCG	594
	GES-R	CCTCTCAATGGTGTGGGT	
bla_*NDM*_	NDM-F	TTGGCCTTGCTGTCCTTG	82
	NDM-R	ACACCAGTGACAATATCACCG	
bla_*iMP*_	FMP-F	GAGTGGCTTAATTCTCRATC	120
	IMP-R	AACTAYCCAATAYRTAAC	
bla_*viM*_	VFM-F	GTTTGGTCGCATATCGCAAC	382
	VFM-R	AATGCGCAGCACCAGGATAG	
bla_*oxA*_	OXA-F	TGTTTTTGGTGGCATCGAT	177
	OXA-R	GTAAMRATGCTTGGTTCGC	

### Multilocus Sequence Typing Analysis

According to the Institute Pasteur MLST and Whole Genome MLST Database,^1^ *K. pneumoniae* MLST was performed to detect seven housekeeping genes (*rpoB*, *gapA*, *mdh*, *pgi*, *phoE*, *infB*, and *tonB*) by PCR. The primers and conditions were posted on the MLST website. The PCR products were sequenced using universal primers: forward primer GTTTTCCCAGTCACGACGTTGTA and reverse primer TTGTGAGCGGATAACAATTTC. The products were sequenced at Sangon Biotech (Shanghai, China). After uploading and comparing the sequencing results, the allele number and sequence type (ST) were obtained.

### Data Collection

For each CR-KP, one carbapenem-susceptible *K. pneumoniae* (CS-KP) was randomly selected. The two groups were matched for sex and age, and separated at the same period (within 30 days). The antimicrobial susceptibility of the two groups was retrospectively analyzed.

### Statistical Analysis

Statistical analyses were performed using WHONET 5.6 and SPSS 22.0 (IBM) software. Descriptive analyses were performed to depict the separation rate of CR-KP, resistance characteristics, and STs. χ^2^ analyses were performed to determine CR-KP distribution, resistance difference between CR-KP and CS-KP, and medical burden. A two-tailed *p*-value of less than 0.05 was considered to be statistically significant.

### Ethics Statement

The human participants involved in this study were in accordance with the Research Ethics Committee of the Guangdong Provincial People’s Hospital, Guangdong Academy of Medical Sciences (KY-Q-2021-149-01). All participants provided oral informed consent. This study did not involve animal-related experiments, and no animal ethical requirements were needed.

## Results

### Distribution of Carbapenem-Resistant *Klebsiella pneumoniae*

**FIGURE 1:**
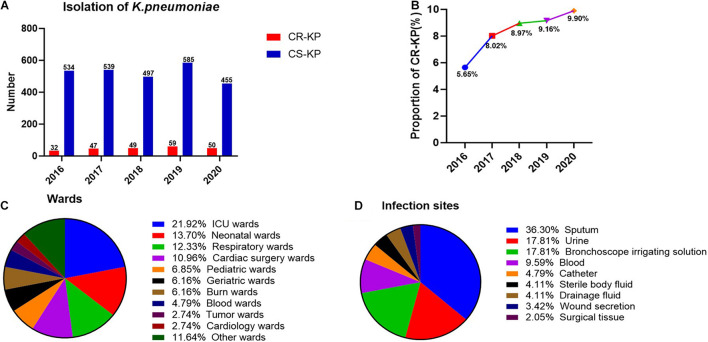
The distribution of CR-KP. **(A)** Isolation of K. pneumoniae in 2016–2020. The x- and y-axes represents years and the number of strains, respectively. **(B)** Proportion of CR-KP in 2016–2020, The x- and y-axes represents years and rates, respectively. **(C)** Distribution of CR-KP in different wards. Colors represent the different wards, the proportion of wards are arranged from top to bottom. **(D)** Distribution of CR-KP in different infection sites. Colors represent the different infection sites, the proportion of infection sites are arranged from top to bottom.

A total of 2,847 isolates were exported from WHONET 5.6, and duplicates were removed, including 146 strains of CR-KP. The separation of CR-KP isolates was increasing except for 2020 because of COVID-19, and the rate was increased from 5.65 to 9.90% (*p* = 0.009) during the study period (Figures 1A,B).

What is more, the distribution of CR-KP in different wards and infection sites showed obvious aggregation: the top 3 wards were ICU (21.92%), neonatal wards (13.70%), and respiratory wards (12.33%), and the top 3 separate samples were sputum (36.30%), urine (17.81%), and bronchoscope irrigating solution (17.81%), and the ICU and neonatal wards have become the key infection areas (Figures 1C,D).

### Antimicrobial Susceptibility Test

As shown in Table 2, CR-KP and CS-KP showed significantly different antimicrobial profiles except for ampicillin; CR-KP showed stronger antimicrobial resistance than the other strains (*p* < 0.001). *K. pneumoniae* is naturally resistant to ampicillin, which is not considered a treatment option. All CS-KP isolates were sensitive to amikacin, cefotaxime, ertapenem, and imipenem, and the resistance rate to piperacillin/tazobactam (2.74%) cefoperazone/sulbactam (6.16%), and tobramycin (6.16%), nitrofurantoin (4.79%) were low. The eight antibacterial agents can be used in the clinical treatment for *K. pneumoniae* infection. The positive rate of CR-KP ESBLs was 7.53% (11/146) and that of CS-KP ESBLs was 50.0% (73/146). This difference was statistically significant (*p* < 0.001). Hence, the mechanisms of carbapenem resistance and ESBLs are different and should be further explored.

**TABLE 2:** The differences in antibacterial profiles between carbapenem-resistant *Klebsiella pneumoniae* (CR-KP) and carbapenem-susceptible *K. pneumoniae* (CS-KP).

Antibacterial agents	Break point (MIC, μ g/ml)		CR-KP (*n* = 146)	CS-KP(*n* = 146)	χ^2^	*P*-value
	R^a^	S^a^				
Piperacillin/Tazobactam	≥128/4	≤16/4	120/146(82.20%)	4/146(2.74%)	185.373	<0.001
Ampicillin/Sulbactam	≥32/16	≤8/4	146/146(100%)	88/146(60.27%)	69.902	<0.001
Ampicillin	≥32	≤8	146/146(100%)	146/146(100%)	/	/
Cefoperazone/Sulbactam^b^	≤15^b^	≥21^b^	138/143(96.50%)	9/146(6.16%)	235.903	<0.001
Cefazolin	≥8	≤2	144/145(99.31%)	84/146(57.53%)	72.408	<0.001
Ceftazidime	≥16	≤4	140/146(95.89%)	17/146(11.64%)	208.430	<0.001
Ceftriaxone	≥4	≤1	143/146(97.94%)	60/146(41.10%)	108.674	<0.001
Cefepime	≥16	≤2	128/146(87.67%)	28/146(19.18%)	137.632	<0.001
Cefotaxime	≥64	≤16	132/146(90.41%)	0/146(0%)	237.264	<0.001
Aztreonam	≥16	≤4	126/146(86.89%)	28/146(19.18%)	131.958	<0.001
Imipenem	≥4	≤1	141/146(96.58%)	0/146(0%)	268.808	<0.001
Ertapenem	≥2	≤0.5	90/91(98.90%)	0/146(0%)	228.621	<0.001
Tobramycin	≥16	≤4	88/146(60.27%)	9/146(6.16%)	96.345	<0.001
Amikacin	≥64	≤16	81/146(55.48%)	0/146(0%)	80.967	<0.001
Gentamicin	≥16	≤4	107/146(73.29%)	30/146(20.55%)	81.529	<0.001
Levofloxacin	≥2	≤0.5	113/146(77.40%)	37/146(25.34%)	79.183	<0.001
Ciprofloxacin	≥1	≤0.25	114/146(78.08%)	45/146(30.82%)	65.740	<0.001
Sulfamethoxazole	≥4/76	≤2/38	85/146(58.22%)	60/146(41.10%)	8.562	0.003
Nitrofurantoin	≥128	≤32	36/80(45.00%)	7/146(4.79%)	54.225	<0.001
Polymyxin B	≥4	≤2	1/37(2.70%)	0/37(0%)	1.014	0.314
ESBL	/	/	11/146(7.53%)	73/146(50.00%)	64.243	<0.001

*^*a*^K, resistance; a, sensitivity.*

*^*b*^Cefoperazone/Sulbactam was quantified through the K-B method; ≥ 21 mm is sensitivity and ≤ 15 mm is resistance.*

### Carbapenemase Phenotypic Tests and Antimicrobial Resistance Genes

**FIGURE 2:**
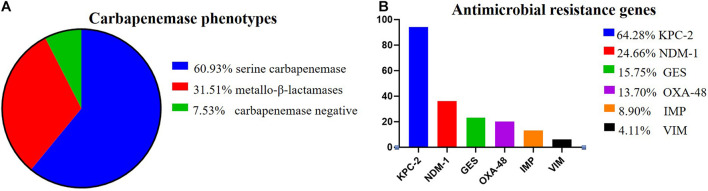
The proportion of carbapenemase phenotypes and antimicrobial resistance genes in CR-KP. **(A)** Proportion of carbapenemase phenotypes. Colors represent the different carbapenemase, which are arranged from top to bottom. **(B)** Proportion of antimicrobial resistance genes. Colors represent the different resistance genes, which are arranged from top to bottom. The x- and y-axes represent genes and the number of isolates, respectively.

Among the 146 CR-KP isolates, 7.53% (11/146) were mCIM negative, indicating that the number of CR-KP with carbapenemase negative was 7.53%. In addition, 92.47% (135/146) were mCIM positive, including 34.07% (46/135) that were eCIM positive, indicating that the numbers of CR-KP producing metallo-β-lactamases were 31.51% (46/146), and CR-KP producing serine carbapenemase were 60.96% (89/146, Figure 2A).

According to the detection results of antimicrobial resistance genes, 94 isolates (64.38%) contained KPC, 23 isolates (15.75%) contained GES, 36 isolates (24.66%) contained NDM, 13 isolates (8.90%) contained IMP, 6 isolates (4.11%) contained VIM, and 20 isolates (13.70%) contained OXA (Figure 2B). Among them, 77 strains had only one gene, 57 strains had two genes, only 1 strain had three genes, and 11 strains had no resistance genes. Interestingly, we found a new double genotype strain and a triple genotype strain for the first time: IMP + GES and KPC + NDM + VIM. These two polygenic strains contained both serine enzyme and metalloenzyme genes, but all the phenotypes were metalloenzyme indicating that metalloenzyme is usually the first choice for CR-KP resistance. What is more, all the ST54 of metalloenzyme type contain IMP, and all the ST45, ST37, and ST76 contain OXA. For enzyme-producing CR-KP, the non-conformity rate of carbapenemase phenotypes and genes reached 17.04% (23/135); 22 isolates produced metallo-β-lactamase while containing KPC or GES or OXA. Only one isolate produced serine carbapenemase while containing IMP and GES, which suggested that when a strain contains both metalloenzyme and serine enzyme genes, the enzyme-producing phenotype is usually metalloenzyme producing.

### Multilocus Sequence Typing

**FIGURE 3:**
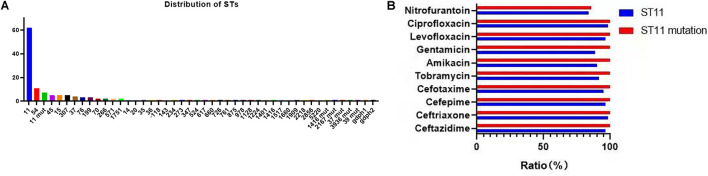
The MLST results of CR-KP. **(A)** The distribution characteristics of STs. The x- and y-axes represents different STs and the number of ST isolates, respectively. **(B)** The differences in antibacterial profiles between ST 11 and ST 11-mutation. The x- and y-axes represents rate of antibacterial resistance and different antibacterials, respectively.

A total of 39 STs were found in 146 CR-KP isolates (Figure 3A). ST11 was the most prevalent type, accounting for 42.47% (62/146), followed by ST54 (7.53%), ST11 mutation (4.79%), ST45/ST15/ST307 (3.42%), ST37 (2.74%), and ST76/ST199; the following ST type accounted for 1.37%: ST70, ST266, ST571, and ST1751; and the remaining ST type accounted for 0.68% (1/146). Moreover, all ST11 isolates contained KPC, and almost all ST11 produced serine carbapenemase (96.77%, 60/62). Of note, the two ST11 isolates that produced metallo-β-lactamases, which contain KPC and NDM, were first found (Table 3) and are resistant to all antibacteria.

**TABLE 3:**
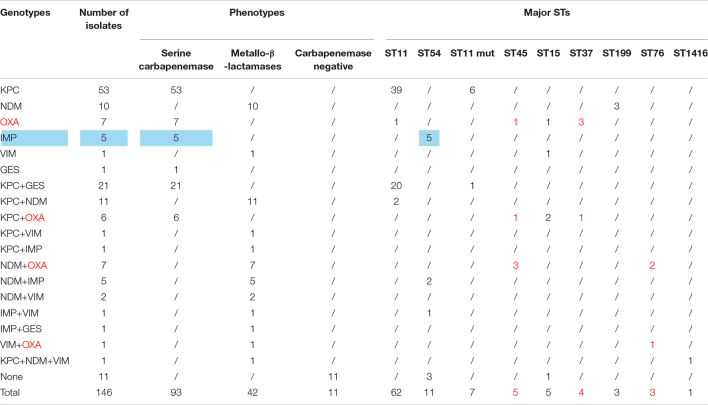
Association between genotype, phenotype and STS.

*Blue, all the ST54 of metalloenzyme type contains IMP; Red, all the ST45, ST37 and ST76 contain OXA.*

The mutation of ST11, as the main epidemic type, should be paid increasing attention. Four types of ST11 allele mutations were found, including seven strains, and four isolates had the same mutation site: their rpoB was mutated from sequence 1 to sequence 146, ST11-mutation (*rpoB 1*–146). As shown in Figure 3B, ST11 isolates were highly resistant to ceftazidime (96.8%), amikacin (90.3%), ciprofloxacin (98.4%), cefotaxime (95.2%), cefepime (96.8%), gentamicin (88.7%), levofloxacin (96.8%), nitrofurantoin (83.9), and tobramycin (92.0%), while all the ST11 mutation (*rpoB* 1–146) isolates were resistant to the 10 antibacterial agents, which means that ST11 mutation (*rpoB* 1–146) was resistant to all antibacteria. In addition, the other three mutants, *rpoB 1*–15, *infB* 3–148, and *infB* 3–80, were also resistant to all antibacteria except for *infB* 3–148, which was sensitive to nitrofurantoin (Table 4). Of note, all the four mutants produced serine carbapenemase and contained KPC indicating that the prevalent strain, ST11, has a serious consequence of mutation, which will lead to an outbreak with the fully resistant, probably.

**TABLE 4:** Characteristics of ST11 and the four mutants.

STs	Mutation	Number of isolates	Phototypes	Genotypes	Antibacterial resistance (%)^a^									
					Ceftazidime	Ceftriaxone	Cefepime	Cefotaxime	Tobramycin	Amikacin	Gentamicin	Levofloxacin	Ciprofloxacin	Nitrofurantoin
ST11	/	60	Serine carbapenemase	KPC, GES, OXA	96.67%	98.33%	96.67%	95.00%	91.67%	90.00%	88.33%	96.67%	98.33%	83.33%
	/	2	Metallo-(J-lactanuses	KPC, NDM	R^b^	R	R	R	R	R	R	R	R	R
	rpoB 1–146	4	Serine carbapenemase	KPC	R	R	R	R	R	R	R	R	R	R
ST11	rpoB 1–15	1	Serine carbapenemase	KPC, GES	R	R	R	R	R	R	R	R	R	R
Mutation	infB 3–148	1	Serine carbapenemase	KPC	R	R	R	R	R	R	R	R	R	S^b^
	infB 3–80	1	Serine carbapenemase	KCP	R	R	R	R	R	R	R	R	R	R

*^*a*^Expect tor the 10 antibacterials in the table, all other antibacterials were resistant.*

*^*b*^R, all isolates were resistant; S, all isolates were sensitive.*

**FIGURE 4:**
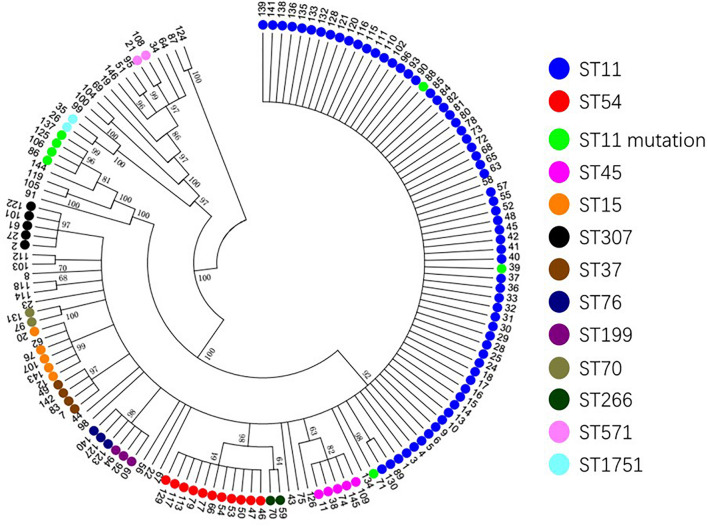
The evolutionary tree of CR-KP. Taking 50% as the cut-off value, different branches represent an evolutionary population. The number represents different samples, and colors represent different STs.

Based on the evolutionary tree analysis (Figure 4), ST11 and the three mutants (39, 71, 88) were in the same branch and had no obvious difference, while ST11-mutation (*rpoB 1*–146) isolates were in an independent separate branch far away (86,106,125,137) indicating that ST11 mutation (*rpoB 1*–146) could be a novel type. The antibacterial profiles, virulence, pathogenicity, and transmission of this mutation may have some unpredictable changes. Hence, ST11 mutation (*rpoB 1*–146) isolate could be a potential novel superbug. Overall, ST11 mutation (*rpoB 1*–146) isolates were the main direction of the future evolution of ST11. This fully resistant, highly pathogenic, and highly transmissible CR-KP with ST11-mutation (*rpoB 1*–146) could become a potential superbug, which can cause incurable infectious diseases and seriously threaten human health.

## Discussion

CR-KP infection has become a major global public health problem. The widespread use and unreasonable application of antibacterial agents are important factors for the increase in the prevalence of CR-KP. In this study, the separation rate of CR-KP increased from 5.65 to 9.90% (*p* = 0.009) from 2016 to 2020. The result is consistent with CARSS, and the detection rate of CR-KP in China increased from 6.4 to 10.1%, from 2014 to 2018 (China Antimicrobial Resistance Surveillance System, 2020). The surveillance result of the European CDC of the European Center for Disease Prevention and Control indicated that the CR-KP rate in European countries, especially in southern and southeastern Europe, increased from 2015 to 2018 (European Centre for Disease Prevention Control, 2019). As early as 2013, the US CDC has listed CR-KP as the highest threat level, that is, Urgent Threat (Centers for Disease Control Prevention, 2013). Thus, how to deal with this urgent threat is a global problem.

Our study showed the following carbapenemase phenotypes: serine carbapenemase (60.96%), metallo-β-lactamases (31.51%), and carbapenemase negative (7.53%). Except for enzyme-producing CR-KP, carbapenemase-negative CR-KP may be caused by many mechanisms, such as extended-spectrum β-lactamases (ESBLs), alterations in penicillin-binding proteins, AmpC cephalosporinases, porin defects (disruption of OmpK35 or/and production of OmpK36 variant), and efflux pumps (Karampatakis et al., 2016; Logan and Weinstein, 2017). The carbapenemase genotypes were KPC (64.38%), NDM (24.66%), GES (15.75%), OXA (13.70%), IMP (8.90%), and VIM (4.11%). For enzyme-producing CR-KP, the non-conformity rate of carbapenemase phenotypes and genes reached 17.04% (23/135). This inconsistency between genotype and phenotype has been reported; three carbapenem genes (blaKPC or blaVIM) were positive with high expression of AmpC in six carbapenemase-negative CR-KP isolates (Li et al., 2019). This phenomenon may be caused by genetic mutation; for example, ST258 contains a Tn4401 variant (Tn4401d), which excises partial KPC fragment, ISKpn7, and partial *tnpA* fragment, resulting in KPC function loss and phenotypic changes (Chen et al., 2012). In addition, the positive rate of CS-KP ESBLs (7.53%) was significantly higher than that of CR-KP ESBLs (50.0%) because of the choice of environmental pressure. Most CR-KP isolates contain carbapenemase-related genes, which can effectively resist antibacterial agents, in contrast to CS-KP. Other methods are needed to resist antibacterial pressure. For example, a premature stop codon at OmpK35 and L3 alterations in OmpK36 resulted in low OmpK expression or OpmC break; or β-lactamases with ceftazidime caused MDR *K. pneumoniae*, except CR-KP (Castanheira et al., 2020).

Many factors could lead to the formation of CR-KP, especially the production of carbapenemase, failing most antibacterial agents. In China, the predominant CR-KP isolates contained KPC and produced serine carbapenemase (Yang et al., 2021). Similarly, in the present study, ST11 is with serine carbapenemase (97.75%, 87/89) and KPC gene (100%). Meanwhile, some local outbreaks of CR-KP with NDM and OXA genes were reported worldwide (Guducuoglu et al., 2018; Solgi et al., 2020). Analysis of strains from Guangdong indicated many CR-KPs with NDM (24.66%) and OXA (13.70%). NDM and OXA can spread horizontally among different strains and can be passed on to the next generation, thereby causing outbreaks (Guducuoglu et al., 2018). Thus, strains that easily cause outbreaks should be given attention in Guangdong. Several studies show that OXA has gradually increased in CR-KP (Pitout et al., 2019; Moussa et al., 2020), while all ST45, ST37, and ST76 isolates contained OXA. This finding is worrisome considering that ST45, ST76, and ST37 can potentially cause outbreaks of hospital infection. Moreover, two polygenic strains were first found: IMP + GES and KPC + NDM + VIM, but all the phenotypes were metalloenzyme, indicating that metalloenzyme was usually the first choice for CR-KP resistance.

MLST analysis revealed the global trend of CR-KP. ST258 is dominant in Europe and the United States, while ST11 is dominant in East Asia (Berglund et al., 2019). ST11 is also a predominant area in China (Li et al., 2019; Huang et al., 2021). In the present study, ST11 was the most prevalent type, followed by ST54 (7.53%), and ST11 mutation (4.79%). ST54 mainly carries IMP and produces metalloenzyme, which was not effectively treated by ceftazidime/averbactam, a new antibacteria used to treat CR-KP with serine carbapenemase, indicating that the epidemic of the ST54 will cause great treatment problems. In addition, the ST54 has become the second most prevalent strain after ST11, so it is worthy of attention. Currently, ST11 is widely distributed worldwide, for example, the first case of ST11 with KPC-3 was reported in Latin America (Garcia-Fulgueiras et al., 2019), and ST11 with NDM broke out in Poland (Baraniak et al., 2019). In the present work, ST11 with KPC and NDM was found; this isolate can be spread horizontally through plasmids and is a threat to potential outbreaks. From the perspective of allele mutation, ST11 and ST258 are only different from *tonB*; the allele *tonB* is 4 in ST11 and 79 in ST258 (Institut Pasteur MLST and Whole Genome MLST Databases, 2016). Interestingly, this study reported for the first time another allelic mutation in ST11, namely, rpoB was mutated from sequence 3 to sequence 146, ST11 mutation (*rpoB 1*–146). As the evolutionary tree shows, ST11 mutation (*rpoB 1*–146) isolates were in a separate branch from ST11. ST11 mutation (*rpoB 1*–146) isolates were resistant to all 19 antibacterial agents in the antibacterial profiles of *K. pneumoniae*, a potential superbug. ST11 isolated from horses has high chromosomal homology to ST11 clinical strains including ST11 KPC-producing WCHKP020098; KPC is located on a novel F33: A-:B-non-conjugative MDR plasmid (Wang et al., 2020). All ST11-mutation (*rpoB 1*–146) isolates contained KPC and produced serine carbapenemase. Thus, this novel, fully resistant, highly pathogenic, and highly transmissible CR-KP isolate may become the predominant strain after ST11 and ST258. This isolate may even cause incurable infectious diseases and seriously threaten human health.

This study has several limitations. First, 237 CR-KP strains were recorded in WHONET 5.6 because of improper storage or preservation. Only 146 strains were successfully recovered and used as research objects, which may partially bias the results. Second, the ST11 mutation (*rpoB 1*–146) isolate, the novel type, was only found from the MLST analysis. The reasons for its full resistance as well as the strength of its virulence and invasiveness have not yet been elucidated; multi-omics sequencing analysis for ST11 variants will be our next research focus.

## Conclusion

In conclusion, the CR-KP infection rate has increased. The predominant CR-KP isolates contained KPC and produced serine carbapenemase, and ST11 was the most prevalent type. ST11 mutation (rpoB 1–146), a novel type with full resistance, has become a potential superbug and a potential super bacterium, which deserves further attention.

## Data Availability Statement

The original contributions presented in the study are included in the article/supplementary material, further inquiries can be directed to the corresponding author/s.

## Ethics Statement

The studies involving human participants were reviewed and approved by the human participants involved in this study were in accordance with Research Ethics Committee of the Guangdong Provincial People’s Hospital, Guangdong Academy of Medical Sciences (KY-Q-2021-149-01). Written informed consent for participation was not required for this study in accordance with the national legislation and the institutional requirements.

## Author Contributions

YZ, YL, BG, and TH designed this study and were in charge of the data collection, MIC testing on the samples, and also contributed to the revision. YZ, YL, NZ, SL, and TH collaborated in drafting the manuscript. YZ conducted the initial analyses with feedback collected from DZ, XH, JZ, QD, YS, and SL. All authors contributed to the article and approved the submitted version.

## Conflict of Interest

The authors declare that the research was conducted in the absence of any commercial or financial relationships that could be construed as a potential conflict of interest.

## Publisher’s Note

All claims expressed in this article are solely those of the authors and do not necessarily represent those of their affiliated organizations, or those of the publisher, the editors and the reviewers. Any product that may be evaluated in this article, or claim that may be made by its manufacturer, is not guaranteed or endorsed by the publisher.

## Footnotes

1
https://bigsdb.pasteur.fr/klebsiella
